# Self-Reported Mental Health History and Self-Reported Coping Behaviours in Biomedicine Students—Exploring Associations with Healthy Lifestyle and Resilience

**DOI:** 10.3390/ejihpe16070089

**Published:** 2026-06-29

**Authors:** Tina Vilovic, Josko Bozic, Marko Kumric, Roko Santic, Josip Vrdoljak, Marion Tomicic, Marko Rada, Edvard Kamsigovski, Mariana Radovic, Marino Vilovic

**Affiliations:** 1 Department of Family Medicine, University of Split School of Medicine, Soltanska 2A, 21000 Split, Croatia; tvilovic@mefst.hr (T.V.); marion.tomicic@mefst.hr (M.T.); ravnatelj@dz-sdz.hr (M.R.); 2 Department of Family Medicine, Split-Dalmatia Health Center, Kavanjinova 2, 21000 Split, Croatia; 3 Department of Pathophysiology, University of Split School of Medicine, Soltanska 2A, 21000 Split, Croatia; josko.bozic@mefst.hr (J.B.); marko.kumric@mefst.hr (M.K.); roko.santic@mefst.hr (R.S.); josip.vrdoljak@mefst.hr (J.V.); edvardk997@gmail.hr (E.K.); mariana.radovic8@gmail.com (M.R.); 4 Laboratory for Cardiometabolic Research, University of Split School of Medicine, Soltanska 2A, 21000 Split, Croatia

**Keywords:** mental health disorders, biomedical students, medical students, self-reported mental health disorder history, coping behaviours, resilience, exhaustion, disengagement, healthy lifestyle, FANTASTIC Lifestyle Questionnaire, cross-sectional study

## Abstract

Background: Mental health disorders (MHDs) are an important concern among biomedical students, but estimates vary depending on whether studies assess confirmed diagnoses, perceived problems or current symptoms. This study assessed the presence of self-reported MHD history, operationalised as a heterogeneous combined outcome that included either a confirmed MHD diagnosis or a subjective perception of having or having had an MHD without official confirmation, and examined its associations with self-reported coping behaviours, resilience, study-related exhaustion and disengagement symptoms and healthy lifestyle adherence. Methods: A cross-sectional online survey was conducted among students from Medicine, Dental Medicine and Pharmacy programmes. Of 936 eligible students, 520 completed the survey (55.6%). The survey assessed self-reported MHD history and coping behaviours, while standardised questionnaires measured resilience, exhaustion and disengagement symptoms and healthy lifestyle adherence. Analyses included descriptive statistics, group comparisons, Spearman correlations, false-discovery-rate correction for exploratory coping-behaviour analyses and age- and gender-adjusted logistic regression. Results: Overall, 159 students (30.6%) had self-reported MHD history present, including 32 (6.2%) with a confirmed diagnosis and 127 (24.4%) with a subjectively perceived MHD without official confirmation. Exhaustion/disengagement scores negatively correlated with resilience and lifestyle scores, while resilience positively correlated with lifestyle scores (all *p* < 0.001). In adjusted logistic regression, higher resilience (OR = 0.539, 95% CI = 0.389–0.747, *p* < 0.001) and lifestyle scores (OR = 0.951, 95% CI = 0.929–0.974, *p* < 0.001) were associated with lower odds of self-reported MHD history being present. Conclusions: Self-reported MHD history was common among respondents and was associated with lower resilience, poorer lifestyle adherence and higher study-related exhaustion and disengagement symptoms. Because the outcome was heterogeneous and the design was cross-sectional, findings should be interpreted as associations rather than clinically verified prevalence estimates or causal effects.

## 1. Introduction

Mental health disorders (MHDs) are an important public health concern among university students, and students in biomedical programmes may face additional pressures related to dense curricula, frequent examinations, clinical exposure, competitive environments, long study hours and expectations regarding future professional performance (Bashir et al., 2020; Ghozy et al., 2022; Nair et al., 2023; Srivastava et al., 2020). Medicine, Dental Medicine and Pharmacy students are future healthcare professionals, and their educational pathway can combine academic, clinical, laboratory and professional demands. This makes biomedical students a relevant population for examining self-reported mental health history, help-seeking and stress-related behaviours.

Comparisons across studies are complicated because mental health outcomes are operationalised in different ways, including clinically confirmed diagnoses, self-reported diagnoses, current symptoms, screening-based distress and subjective perceptions of having a mental health problem. This distinction is particularly important in student populations because stigma, confidentiality concerns and limited help-seeking may reduce the proportion of students with formally confirmed diagnoses (Fletcher et al., 2020; Gold et al., 2016; Villatoro et al., 2022). Therefore, studies using self-reported mental health history should clearly distinguish clinically confirmed diagnoses from perceived but unconfirmed problems.

Resilience, coping behaviours, healthy lifestyle adherence and burnout symptoms are conceptually related but distinct constructs. Resilience refers to the capacity to recover from stress and adversity (Liu et al., 2018; Smith et al., 2008). Healthy lifestyle adherence includes behaviours such as nutrition, physical activity, sleep, stress management and social connection, which may be associated with psychological well-being (Lianov, 2021; Ruiz-Hernández et al., 2022; Velten et al., 2018). Coping behaviours represent the actions students report using under stress, and these may include both adaptive and potentially maladaptive responses (Baqutayan, 2015; Martínez et al., 2020; Sattar et al., 2022). In line with Lazarus and Folkman’s stress-and-coping framework, coping behaviours were considered as self-reported behavioural responses to stress (Lazarus & Folkman, 1984). However, because coping was assessed using a study-specific checklist, this framework was used only as conceptual background and was not formally tested. Burnout symptoms, in student samples, are best interpreted as study-related exhaustion and disengagement rather than a clinically verified occupational disorder.

The expected pattern is that lower resilience and poorer lifestyle adherence may co-occur with more self-reported mental health problems, while higher exhaustion and disengagement symptoms may reflect greater stress-related strain. However, these relationships may also operate in the opposite direction: students with mental health difficulties may have lower resilience scores, poorer lifestyle routines or higher exhaustion and disengagement symptoms because of their psychological burden. A cross-sectional design can therefore identify associations among these constructs, but it cannot establish whether one construct causes another.

In the present study, exhaustion and disengagement symptoms were assessed using the Oldenburg Burnout Inventory (OBI). Although the OBI was originally developed to assess burnout-related symptoms in occupational contexts, OBI scores were not used to diagnose occupational burnout in this student sample. Rather, they were used to quantify exhaustion and disengagement symptoms that may occur in a demanding study context. This interpretation is particularly important because employment status, academic workload and specific educational resources were not directly measured.

Despite the growing literature on mental health in health-profession students, fewer studies have simultaneously examined self-reported MHD history, self-reported coping behaviours, resilience, exhaustion and disengagement symptoms and lifestyle adherence in one biomedical-student sample. This combined assessment may help identify patterns that are relevant for future longitudinal and interventional research, while avoiding causal interpretation of cross-sectional findings.

Therefore, the primary aim of this study was to assess the proportion of students with self-reported MHD history present in a biomedicine student population. Additionally, we aimed to investigate associations between self-reported MHD history, self-reported coping behaviours, resilience, healthy lifestyle adherence and study-related exhaustion and disengagement symptoms.

## 2. Materials and Methods

### 2.1. Participants and Procedures

This cross-sectional study was conducted through an anonymous online survey among biomedical students attending the University of Split School of Medicine in Croatia. Eligible students were enrolled in Medicine, Dental Medicine and Pharmacy study programmes, with all study years included. During the academic year 2021/2022, the eligible biomedical student population comprised 936 students. The minimum required sample size was calculated before data collection using the SurveyMonkey^®^ sample size calculator, assuming a 95% confidence level, a 5% margin of error, a conservative expected response distribution/expected prevalence of 50% and finite population correction for the eligible population of 936 students. This yielded a minimum required sample of 273 students. A total of 520 students completed the survey, corresponding to a completion proportion of 55.6% and exceeding the minimum required sample. The final sample included 268 Medicine, 169 Dental Medicine and 83 Pharmacy students. The online link to the Google Forms^®^ survey was distributed to all eligible students through the official e-mail addresses of student representatives for individual study years and programmes, while data were collected during the COVID-19 pandemic period, between December 2021 and March 2022.

All relevant information was presented to students in invitation e-mails and in the introductory section of the survey, and potential questions could be asked via the included e-mail address. Participation was voluntary and anonymous. Survey submission was considered electronic informed consent, and this was emphasised to students in the survey introduction. No names, student identification numbers or other directly identifying personal data were requested, and students could leave the questionnaire before submission. The investigation was carried out in accordance with the ethical standards of the Declaration of Helsinki and was approved by the Ethics Committee of the University of Split School of Medicine (No.: 2181-198-03-04-21-0089).

### 2.2. Measures

#### Study-Specific Survey, Pilot Testing and Mental Health Disorder History

The study-specific survey was developed at the Department of Family Medicine, University of Split School of Medicine, after literature review on COVID-19, mental health, resilience and exhaustion/disengagement symptoms among biomedical students. Methodological examples from previous studies informed the first section assessing baseline and mental health-related information (Gold et al., 2016; Kang et al., 2020; Lipson et al., 2021; Shechter et al., 2020). The second section consisted of standardised questionnaires measuring resilience, study-related exhaustion and disengagement symptoms and healthy lifestyle adherence. Both sections were administered, completed and submitted at the same time via Google Forms^®^. Before the main distribution, the survey was pilot-tested among 20 students from different study years and programmes who were invited separately to evaluate item comprehensibility and completion time. Pilot participants did not report problems with comprehensibility, and the average completion time was approximately 12 min. Pilot responses were used only for feasibility assessment and were not included in the final analytical sample of 520 students.

The first section consisted of 10 items, including age, gender, study programme and study year data. Regarding MHD history, students were asked whether they had ever had, or currently had, a confirmed MHD diagnosis, including anxiety, depression, post-traumatic stress disorder, addiction or another condition they considered relevant. They were also asked whether they subjectively considered that they had, or had had, an MHD that was never officially confirmed. In the main analyses, self-reported MHD history was considered present if students reported either a confirmed MHD diagnosis or a subjectively perceived MHD without official confirmation. The decision to combine these groups was made because the number of students with confirmed diagnoses was small (*N* = 32), which limited statistical power for the main regression analyses, and because perceived but unconfirmed problems may be relevant in student populations with possible stigma or low help-seeking. However, this combined category is heterogeneous and should not be interpreted as a clinically verified prevalence of MHDs. Sensitivity analyses were therefore performed by separating students with confirmed diagnoses from students with subjectively perceived but unconfirmed MHD history. Two further items asked whether the MHD emerged during the COVID-19 pandemic and whether students with a self-reported MHD history present had sought professional help. Finally, this section assessed self-reported coping behaviours. Information on subjective self-assessment of stress-coping ability was collected, after which students selected from 11 behaviours they reported using when under stress. A similar construction was previously used in a family physician population (Vilovic et al., 2022).

### 2.3. Brief Resilience Scale

Resilience was assessed using the Brief Resilience Scale (BRS), which measures the ability to bounce back from stressful situations and experiences (Smith et al., 2008). The BRS consists of 6 statements answered on a 5-point Likert scale. The final score is calculated as the arithmetic mean of all items, with higher scores indicating greater resilience. Scores from 1.0 to 2.9 indicate low resilience, 3.0 to 4.3 normal resilience and 4.31 to 5.0 high resilience. The BRS has been validated in Croatian, with adequate internal consistency (coefficient α = 0.82) (Slišković et al., 2018). In the present study, coefficient α was 0.83.

### 2.4. Study-Related Exhaustion and Disengagement Symptoms

Study-related exhaustion and disengagement symptoms were assessed using the Oldenburg Burnout Inventory (OBI), which includes 16 items and two 8-item subscales: exhaustion (OBI-E) and disengagement (OBI-D) (Bakker & Demerouti, 2007). Items are scored from 1 to 4 after appropriate reverse coding, so the total OBI score ranges from 16 to 64, with higher scores indicating more pronounced exhaustion and disengagement symptoms. Because burnout is conventionally conceptualised as a work-related phenomenon, OBI scores in this student sample were interpreted as study-related exhaustion and disengagement symptoms rather than as a formally diagnosed occupational burnout disorder. The OBI has been validated in Croatian, with good internal consistency for OBI-E (coefficient α = 0.84) and OBI-D (coefficient α = 0.76) (Slišković et al., 2018). In our study, coefficient α was 0.84 for the total OBI score, 0.80 for OBI-E and 0.72 for OBI-D.

### 2.5. FANTASTIC Lifestyle Questionnaire

Physical, social and psychological facets of a healthy lifestyle were evaluated using the FANTASTIC Lifestyle Questionnaire (FLQ), originally developed by Wilson et al. (Wilson et al., 1984). The questionnaire comprises 25 questions about behaviour during the past month and includes nine domains: F—family/friends, A—activity, N—nutrition, T—tobacco/toxins, A—alcohol, S—sleep/seatbelt/stress/safe sex, T—type of behaviour, I—insight and C—career. Most items offer five response options on a Likert scale, while two are dichotomous. The total score ranges from 0 to 100 points, with higher scores indicating a more health-conscious lifestyle. Based on the final score, participants were classified into five categories: 0–34 points, “needs improvement”; 35–54 points, “fair”; 55–69 points, “good”; 70–84 points, “very good”; and 85–100 points, “excellent” (Horaist & Watson, 2024). The questionnaire has shown good reliability in previous studies, including student populations, and was translated into Croatian using the back-translation technique (Murillo-Llorente et al., 2022). In our study, coefficient α was 0.74. The authors have permission to use BRS, OBI and FLQ instruments from the copyright holders.

### 2.6. Statistical Analysis

We used the MedCalc program 19.1.2. (MedCalc Software, Ostend, Belgium) for statistical analysis. Categorical data were presented as N (%), and the chi-squared test was used to assess differences between categorical variables. The distribution of continuous data was evaluated using the D’Agostino–Pearson test, which indicated non-normal distributions. Therefore, continuous variables were presented as median values with interquartile ranges, and differences were assessed using the Mann–Whitney U test or the Kruskal–Wallis test with post hoc Conover analysis, where appropriate. *p* values in Table 1 and Table 2 are unadjusted descriptive comparisons. For exploratory analyses of the 11 individual self-reported coping behaviours in relation to self-reported MHD history, *p* values were additionally corrected using the Benjamini–Hochberg false discovery rate (FDR) procedure, and FDR-adjusted q values are reported. Non-parametric ANCOVA using the Quade method was used as a supplementary adjusted analysis to evaluate whether differences in OBI, FLQ and BRS scores across gender, study-year category and study programme remained present after adjustment for age and the other questionnaire scores, where appropriate. Multivariable logistic regression was conducted to examine factors associated with self-reported MHD history being present. Age and gender were retained a priori as adjustment variables, while BRS, FLQ and OBI scores were evaluated as continuous candidate variables using a forward selection approach. Variables were retained in the final model at *p* < 0.05. Results are presented as odds ratios (ORs), 95% confidence intervals (95% CIs) and *p* values. Goodness of fit was assessed using the Hosmer–Lemeshow test. Sensitivity analyses separately compared students with confirmed MHD diagnoses against those without self-reported MHD history and students with subjectively perceived but unconfirmed MHD history against those without self-reported MHD history. Because of the cross-sectional design, regression results were interpreted as associations rather than causal predictors. Statistical significance was considered at *p* < 0.05.

## 3. Results

In this study, 520 of 936 eligible biomedicine students completed the survey, corresponding to a completion proportion of 55.6%. The majority of respondents were women (*N* = 405). Most respondents were students of the Medicine programme (*N* = 268), followed by Dental Medicine (*N* = 169) and Pharmacy (*N* = 83), with all study years included. A total of 159 students (30.6%) had self-reported MHD history present, including 32 students (6.2% of the total sample) with a confirmed MHD diagnosis and 127 students (24.4% of the total sample) with a subjectively perceived MHD without official confirmation. Self-reported MHD history was more frequently present among women than men (33.1 vs. 21.7%; *p* = 0.019) (Table 1). Moreover, among students with self-reported MHD history present, most reported that the disorder started before the onset of the COVID-19 pandemic (*N* = 119; 74.8%), while a minority had actively sought professional help (*N* = 44; 27.7%).

Furthermore, most student participants reported subjectively good stress-coping ability (*N* = 380; 73.1%), with significantly more positive answers from men when compared with women (85.2 vs. 69.6%; *p* < 0.001). Analysis of self-reported coping behaviours showed that the most frequently reported behaviours were “listening to music” (*N* = 364; 70.0%) and “communication with friends” (*N* = 212; 40.8%), while “drinking alcoholic drinks” and “working out” were more frequently reported by men than women (13.9 vs. 7.9%; *p* = 0.048 and 52.2 vs. 35.3%; *p* = 0.001, respectively). Finally, “spending time with family” was more frequently reported by women (39.0 vs. 26.1%; *p* = 0.011). See Table 1 for the detailed analysis of baseline characteristics and self-reported coping behaviours according to gender. These *p* values are unadjusted descriptive comparisons.

**Figure 1:**
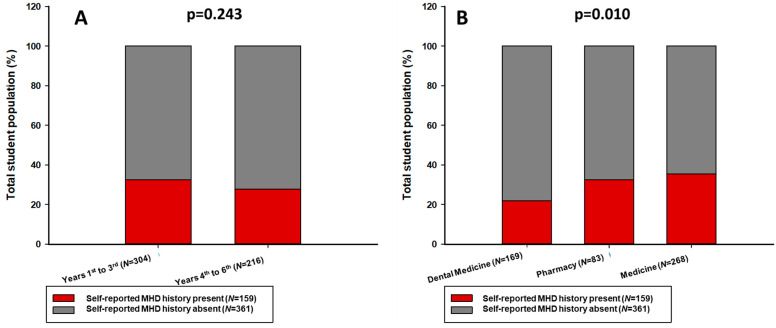
Distribution of self-reported MHD history according to (**A**) year of study and (**B**) study programme. Bars show the percentage of students with self-reported MHD history present and absent. Self-reported MHD history was considered present if students reported a confirmed MHD diagnosis or subjectively perceived MHD without official confirmation. MHD, mental health disorder; *p* values are from chi-square tests.

Further analysis of self-reported MHD history was conducted according to year and type of biomedicine study programme. Results showed no statistically significant difference according to study year (years 1–3: 32.6%; years 4–6: 27.8%; *p* = 0.243) (Figure 1A). According to the study programme, self-reported MHD history was most frequently present among Medicine students (35.4%), followed by Pharmacy students (32.5%) and Dental Medicine students (21.9%; *p* = 0.010) (Figure 1B).

**Figure 2:**
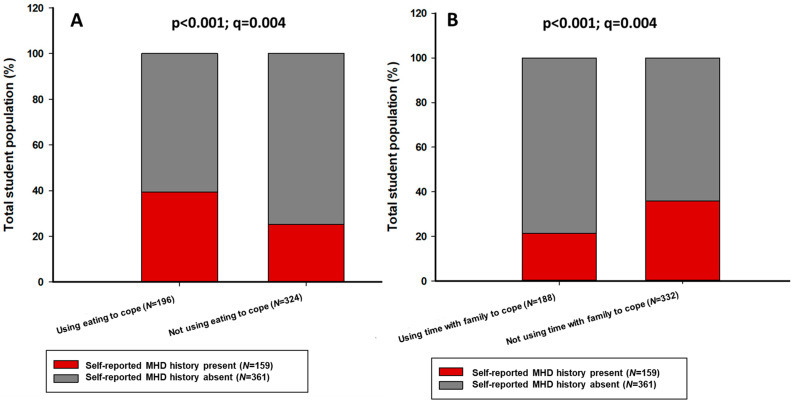
Self-reported coping behaviours and self-reported MHD history: (**A**) using eating to cope and (**B**) using time with family to cope. Bars show the percentage of students with self-reported MHD history present and absent within each behaviour group. Self-reported MHD history was considered present if students reported a confirmed MHD diagnosis or subjectively perceived MHD without official confirmation. MHD, mental health disorder; *p* values are from chi-square tests, and q values are Benjamini–Hochberg FDR-adjusted values across 11 coping-behaviour comparisons.

Furthermore, analyses of self-reported coping behaviours according to self-reported MHD history showed that a higher percentage of students with self-reported MHD history present was found among those who reported using “eating” to cope (39.3 vs. 25.3%; unadjusted *p* < 0.001; FDR q = 0.004). A lower percentage of students with self-reported MHD history present was found among those who reported “spending time with family” to cope (21.3 vs. 35.8%; unadjusted *p* < 0.001; FDR q = 0.004) (Figure 2). Smoking showed an unadjusted association (40.7 vs. 28.6%; unadjusted *p* = 0.026), but this did not remain statistically significant after FDR correction across the 11 coping-behaviour comparisons (q = 0.094). Other analyses of self-reported coping behaviours according to self-reported MHD history showed no statistically significant differences after correction (q > 0.05). These analyses were exploratory.

Afterwards, we conducted specific analyses of questionnaires used to assess OBI exhaustion/disengagement scores, resilience (BRS) and healthy lifestyle adherence (FLQ). Results showed that BRS and FLQ scores were significantly higher in students who reported good stress-coping ability than in those who reported poor stress-coping ability (*p* < 0.001), while OBI scores were significantly lower (*p* < 0.001). In contrast, students with self-reported MHD history present had significantly lower BRS and FLQ scores and significantly higher OBI scores than students without such history (*p* < 0.001 for all comparisons) (Table 2). Moreover, when considering only students with self-reported MHD history present, higher OBI scores were found in students who sought professional help in comparison with others (44 (38–49) vs. 40 (36–45); *p* = 0.014) (Table 2).

Supplementary adjusted analyses using non-parametric ANCOVA with the Quade method are presented in Supplementary Table S1. These analyses showed selected adjusted differences in questionnaire scores across demographic groups, including associations between gender and both FLQ and BRS scores, study-year category and FLQ score, and study programme and OBI score.

**Figure 3:**
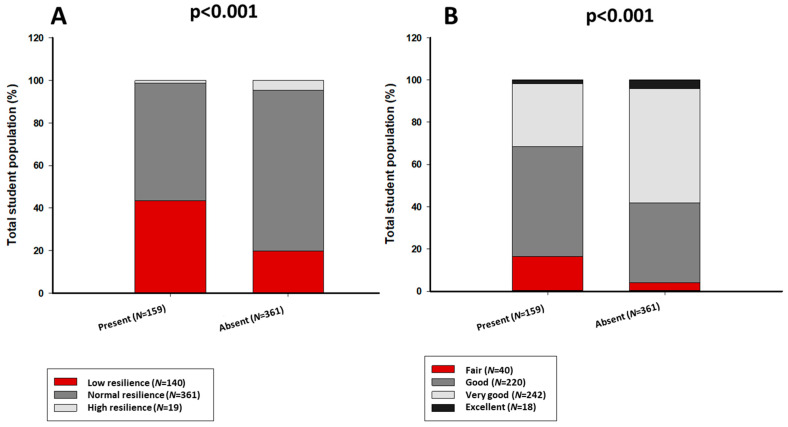
Distribution of (**A**) resilience categories and (**B**) FANTASTIC Lifestyle Questionnaire categories according to self-reported MHD history. Self-reported MHD history was considered present if students reported a confirmed MHD diagnosis or subjectively perceived MHD without official confirmation. BRS, Brief Resilience Scale; FLQ, FANTASTIC Lifestyle Questionnaire; MHD, mental health disorder; *p* values are from chi-square tests.

Also, results showed that most of the population had normal resilience levels (*N* = 361; 69.4%), while 140 students (26.9%) had low resilience and 19 students (3.7%) had high resilience. Significantly more students with self-reported MHD history present had low resilience than those without such history (43.4 vs. 19.7%; *p* < 0.001) (Figure 3A). Moreover, analysis of healthy lifestyle adherence according to self-reported MHD history showed that students with self-reported MHD history present had a higher percentage of fair (15.7 vs. 3.9%) and good lifestyle categories (52.2 vs. 38.0%; *p* < 0.001) (Figure 3B), indicating a distribution shifted toward lower FLQ categories compared with students without self-reported MHD history. Although the FLQ classification includes a “needs improvement” category, no respondent in the final analytical sample was classified in this category.

**Figure 4:**
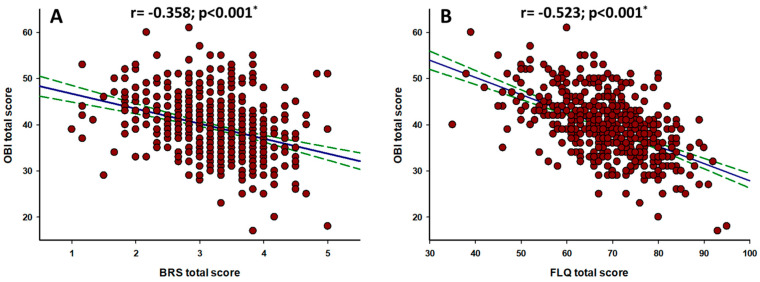
Correlations between OBI scores and (**A**) BRS resilience score and (**B**) FLQ lifestyle score. Dots represent individual respondents, and lines indicate fitted trends with confidence bands. BRS, Brief Resilience Scale; FLQ, FANTASTIC Lifestyle Questionnaire; OBI, Oldenburg Burnout Inventory; Spearman’s rank correlation coefficients and *p* values are shown (*). Blue lines represent correlation coefficient and green lines represent respective 95% confidence intervals.

Further statistical analysis included correlation assessment between scores of the used questionnaires. Significant negative correlations were found between OBI and BRS scores (r= −0.358; *p* < 0.001) (Figure 4A) and between OBI and FLQ scores (r= −0.523; *p* < 0.001) (Figure 4B), while a significant positive correlation was determined between BRS and FLQ scores (r = 0.436; *p* < 0.001).

Finally, multivariable logistic regression was conducted to assess factors associated with self-reported MHD history being present. In the final age- and gender-adjusted model, higher BRS score (OR = 0.539, 95% CI = 0.389–0.747; *p* < 0.001) and higher FLQ score (OR = 0.951, 95% CI = 0.929–0.974; *p* < 0.001) were associated with lower odds of self-reported MHD history being present, while age and gender were not statistically significant (Table 3). OBI score was evaluated as a candidate variable but was not retained in the final model; when entered with age, gender, BRS and FLQ, OBI was not independently associated with self-reported MHD history (OR = 1.026, 95% CI = 0.988–1.065; *p* = 0.176). This may reflect shared variance between OBI-assessed exhaustion/disengagement symptoms, resilience and lifestyle scores. The Hosmer–Lemeshow goodness-of-fit test did not indicate poor model fit for the final logistic regression model (χ^2^ = 5.00, df = 8, *p* = 0.758). Sensitivity analyses that separated confirmed MHD diagnoses from subjectively perceived but unconfirmed MHD history showed the same general direction of associations, although estimates for confirmed diagnoses were less precise because of the small number of confirmed cases (Table 4).

## 4. Discussion

The present cross-sectional study assessed self-reported MHD history, self-reported coping behaviours, resilience, exhaustion/disengagement symptoms and healthy lifestyle adherence among biomedicine students. Overall, 30.6% of respondents had self-reported MHD history present; however, this combined outcome included 32 students with a confirmed diagnosis and 127 students with a subjective perception of having or having had an MHD without formal confirmation. Therefore, this proportion should not be interpreted as the prevalence of clinically verified MHDs, but rather as the proportion of students reporting either confirmed or perceived MHD history. Self-reported MHD history was more frequent among women and students enrolled in the Medicine programme, while only a minority of students with self-reported MHD history present reported seeking professional help. In addition, students with self-reported MHD history present had lower resilience and healthy lifestyle scores, as well as higher exhaustion/disengagement symptom scores. Resilience and healthy lifestyle scores were also associated with self-reported MHD history in the adjusted logistic regression model, while exhaustion/disengagement symptoms showed negative correlations with both resilience and healthy lifestyle adherence.

The proportion of students reporting confirmed or perceived MHD history in our study is consistent with the broader literature indicating a substantial psychological burden among students in health-related disciplines. However, direct comparison with previous studies should be made cautiously because studies differ considerably in their definitions and measurement of mental health outcomes, including clinically confirmed diagnoses, self-reported diagnoses, current symptoms, screening-based psychological distress, depression, anxiety or general mental well-being. For example, Jia et al. (2022), in a meta-analysis including more than 36,000 medical students during the COVID-19 pandemic, reported pooled prevalences of depressive and anxiety symptoms of 37.9% and 33.7%, respectively. Similarly, previous studies have emphasised that medical and biomedical students may be especially vulnerable to mental health difficulties because of high academic demands, clinical responsibilities, competitive environments, long study hours and fear of future professional mistakes (Bashir et al., 2020; Mahmood et al., 2025; Nair et al., 2023; Srivastava et al., 2020). The qualitative study by Mahmood et al. (2025) further supports this interpretation by describing time constraints, academic workload, clinical rotations, limited mentorship, inadequate research training and institutional barriers as factors that may contribute to the high-pressure environment experienced by medical and dental students. The COVID-19 pandemic may have further contributed to psychological burden through changes in teaching modalities, uncertainty about academic progression and concerns related to future work in healthcare settings (Ghozy et al., 2022; Machado et al., 2023; Vilovic et al., 2021). Nevertheless, since most students with self-reported MHD history present in our study reported that their problems had started before the pandemic, the findings suggest that mental health difficulties in this population were not solely pandemic-related.

The higher proportion of self-reported MHD history among female students is also in line with previous literature showing gender differences in the reporting and prevalence of several mental health problems, particularly depression and anxiety (Delgado-Herrera et al., 2024). This difference may reflect a combination of biological, psychological and social factors, but it may also be influenced by differences in willingness to recognise or report mental health difficulties. Cultural and institutional contexts should also be considered, since stigma and attitudes toward mental illness can shape both the reporting of mental health problems and help-seeking behaviour (Fekih-Romdhane et al., 2023; Heim et al., 2017; Villatoro et al., 2022). In the Croatian biomedical education context, possible barriers may include small student cohorts, close links between academic and clinical settings, concerns about confidentiality, normalisation of high stress as part of professional training, limited time because of demanding curricula and uncertainty about where to seek confidential student-orientated support. These explanations remain speculative because barriers to help-seeking were not directly measured in our survey. In our study, only 44 of 159 students with a self-reported MHD history present reported seeking professional help. Therefore, possible explanations such as stigma, limited access to services, perceived lack of time, low perceived need, previous negative experiences or preference for self-management should be examined directly in future Croatian studies. Similar concerns have been described among medical students, who may avoid disclosure or professional support because of concerns about confidentiality, stigma or potential effects on their future careers (Fletcher et al., 2020).

Self-reported coping behaviours showed several relevant patterns. The most frequently reported behaviours were listening to music and communication with friends, while gender differences were observed for some behaviours, including alcohol consumption, working out and spending time with family. After FDR correction for 11 coping-behaviour comparisons, students with self-reported MHD history more frequently reported eating as a coping behaviour, while spending time with family was less frequent among them. The initially observed association with smoking did not remain statistically significant after correction and should therefore not be interpreted as a robust finding. These results are broadly consistent with previous studies suggesting that students use a variety of coping strategies, some of which may be adaptive and others potentially maladaptive (Graves et al., 2021; Palupi & Findyartini, 2019; Sattar et al., 2022). Viewed within Lazarus and Folkman’s stress-and-coping framework, these behaviours may be interpreted as self-reported responses students use when facing stress. However, because coping was assessed with a study-specific checklist rather than a validated coping instrument, these findings should be considered exploratory and hypothesis-generating.

Resilience was lower among students with self-reported MHD history present, and students who reported good stress-coping skills also had higher resilience scores. These findings are consistent with the literature suggesting that resilience is associated with better mental health outcomes and may influence how students respond to academic and psychological stressors (Healy et al., 2023; Lin et al., 2019; Rasheed et al., 2022). In our sample, 26.9% of students had low resilience, which is lower than the 36% reported in the meta-analysis by Chua et al. (2023), which included students from 18 countries. This difference may be related to differences in study populations, measurement approaches, pandemic context and overall methodological heterogeneity. Resilience should not be viewed as a fixed trait only, but rather as a dynamic capacity influenced by individual, social and environmental factors (Liu et al., 2018; Mesman et al., 2021). Previous intervention studies and reviews have suggested that resilience-building programmes may be beneficial in higher education settings (Akeman et al., 2020; Ang et al., 2022; Kunzler et al., 2020). However, because our study was cross-sectional, it cannot determine whether lower resilience contributed to mental health difficulties, whether mental health difficulties reduced resilience, or whether both were influenced by other unmeasured factors.

Healthy lifestyle adherence was also lower among students with a self-reported MHD history present and was positively correlated with resilience, while showing a negative correlation with exhaustion/disengagement symptoms. These findings are in line with studies suggesting that lifestyle-related factors, including physical activity, sleep quality, diet and broader health behaviours, are associated with psychological well-being and resilience (Du et al., 2021; Lancaster & Callaghan, 2022; Nishimi et al., 2022; Velten et al., 2018). Ruiz-Hernández et al. (2022) reported associations between poorer lifestyle habits and psychopathology among university students, while Ghali et al. (2022) found that physical activity was associated with psychiatric disorders among medical students during the COVID-19 pandemic. Nevertheless, the direction of these associations remains uncertain. Students with mental health difficulties may find it harder to maintain healthy routines, while poorer sleep, reduced physical activity, unhealthy eating or substance use may also contribute to psychological vulnerability. Therefore, lifestyle should be interpreted as an associated factor rather than a proven protective factor in the present study.

Study-related exhaustion and disengagement symptoms were also part of the observed pattern of associations. Students with self-reported MHD history present had higher OBI scores, and OBI scores were negatively correlated with resilience and healthy lifestyle adherence. These findings should not be interpreted as evidence of occupational burnout in the strict workplace sense. Rather, they suggest that exhaustion and disengagement symptoms may co-occur with poorer self-reported mental health, lower resilience and poorer lifestyle adherence in a demanding educational context. Because the present study did not measure employment status, academic workload or specific academic resources, these findings should be interpreted cautiously and descriptively.

The main practical implication of this study is that biomedical students may benefit from structured, accessible and non-stigmatising mental health support within the academic environment. The simultaneous assessment of self-reported MHD history, coping behaviours, resilience, exhaustion/disengagement symptoms and lifestyle provides a broader overview of potentially relevant areas for student support. However, the findings should not be interpreted as evidence that resilience training, lifestyle interventions or coping-orientated programmes would necessarily reduce MHD occurrence. Rather, they identify potentially relevant targets that should be further examined in longitudinal and interventional studies. Educational institutions may consider strengthening confidential counselling services, promoting mental health literacy, reducing stigma, encouraging help-seeking and supporting healthier routines, but the effectiveness of such approaches should be formally evaluated.

Several limitations should be acknowledged. First, the cross-sectional design prevents conclusions about causality or temporal direction. Second, the main outcome combined students with confirmed MHD diagnoses and those with subjectively perceived MHD history, making the outcome heterogeneous and not equivalent to clinically verified MHD prevalence. Although sensitivity analyses separated these groups, the confirmed-diagnosis group was small, and estimates were imprecise. Third, MHD history was self-reported, and official medical records or structured clinical interviews were not used. Fourth, the exact timing, duration and severity of mental health problems were not assessed, which limits interpretation of their relationship with current resilience, exhaustion/disengagement symptoms and lifestyle scores. Fifth, this study was conducted at a single university, and participation was voluntary and web-based. Although 520 of 936 eligible students completed the survey, corresponding to a response rate of 55.6%, selection bias and non-response bias cannot be excluded. Sixth, although Lazarus and Folkman’s stress-and-coping framework was used as conceptual background, coping behaviours were assessed with a study-specific checklist rather than a validated coping instrument, and the present study did not formally test this model. Seventh, several potentially important confounders, including socioeconomic status, academic performance, treatment history, current symptom severity, chronic illness, sleep details, academic workload, employment status, individual COVID-19-related impact and pandemic-related stressors, were not assessed. Finally, FDR correction was applied to the exploratory coping-behaviour analyses, but other *p* values in descriptive tables were unadjusted; therefore, statistically significant findings with borderline *p* values should be interpreted cautiously and as hypothesis-generating. Although the OBI was used, the present study did not assess occupational burnout as a clinical or work-related disorder. OBI scores were therefore interpreted only as indicators of study-related exhaustion and disengagement symptoms.

In summary, this study found that self-reported MHD history was common among respondents and was associated with lower resilience, lower healthy lifestyle adherence and higher exhaustion/disengagement symptoms among biomedicine students. The findings are consistent with previous research showing a substantial psychological burden in health-profession students, but they should be interpreted within the limits of a cross-sectional, self-reported survey. Future longitudinal studies should distinguish between clinically confirmed diagnoses, perceived mental health problems and current symptom burden, while also examining barriers to help-seeking and testing whether interventions focused on resilience, support aimed at reducing study-related exhaustion and disengagement, lifestyle support and adaptive coping can improve student mental health outcomes.

## 5. Conclusions

In conclusion, this study found that self-reported MHD history was common among respondents from Medicine, Dental Medicine and Pharmacy programmes. This combined outcome included both confirmed diagnoses and subjectively perceived but unconfirmed MHD history and should therefore not be interpreted as clinically verified MHD prevalence. Self-reported MHD history was associated with lower resilience, lower healthy lifestyle scores and higher exhaustion/disengagement symptoms, while exhaustion/disengagement symptoms were negatively correlated with both resilience and healthy lifestyle adherence. Because this study was cross-sectional, these results should be interpreted as associations rather than evidence of causality, protective effects or intervention effectiveness. Further longitudinal, qualitative and interventional studies are needed to clarify temporal relationships and to examine whether resilience-building, burnout prevention, lifestyle-orientated support and accessible mental health services can improve student well-being.

## Figures and Tables

**Table 1:** Baseline characteristics and self-reported coping behaviours of the study population according to gender.

Parameter	Men (*N* = 115)	Women (*N* = 405)	Total (*N* = 520)	*p* *
Baseline characteristics				
Age (years)	21.0 (20.0–24.0)	21.0 (20.0–23.0)	21.0 (21.0–23.0)	0.104
Study programme				
Medicine	77 (67.0)	191 (47.2)	268 (51.5)	<0.001
Dental Medicine	28 (24.3)	141 (34.8)	169 (32.5)	
Pharmacy	10 (8.7)	73 (18.0)	83 (16.0)	
Year of study				
Years 1st to 3rd	67 (58.3)	237 (58.5)	304 (58.5)	0.961
Years 4th to 6th	48 (41.7)	168 (41.5)	216 (41.5)	
Good self-reported stress-coping ability ^†^	98 (85.2)	282 (69.6)	380 (73.1)	<0.001
Self-reported MHD history present ^‡^	25 (21.7)	134 (33.1)	159 (30.6)	0.019
Self-reported coping behaviours				
Smoking	22 (19.1)	64 (15.8)	86 (16.5)	0.397
Drinking alcoholic drinks	16 (13.9)	32 (7.9)	48 (9.2)	0.048
Working on business/school projects	10 (8.7)	20 (4.9)	30 (5.8)	0.128
Eating food	39 (33.9)	157 (38.8)	196 (37.7)	0.343
Watching television	45 (39.1)	157 (38.8)	202 (38.8)	0.943
Reading	18 (15.7)	62 (15.3)	80 (15.4)	0.928
Religious/spiritual activities	20 (17.4)	101 (24.9)	121 (23.3)	0.091
Communication with friends	37 (32.2)	175 (43.2)	212 (40.8)	0.034
Working out	60 (52.2)	143 (35.3)	203 (39.0)	0.001
Listening to music	82 (71.3)	282 (69.6)	364 (70.0)	0.729
Spending time with family	30 (26.1)	158 (39.0)	188 (36.2)	0.011

Data are presented as N (%) or median (interquartile range); MHD—mental health disorder; * chi-square test or Mann–Whitney U test; *p* values are unadjusted descriptive comparisons; ^†^ subjective self-assessment of stress-coping ability; ^‡^ self-reported MHD history present, defined as confirmed MHD diagnosis or subjectively perceived MHD without official confirmation.

**Table 2:** Resilience, study-related exhaustion/disengagement and healthy lifestyle scores according to investigated parameters.

Parameter	OBI Score	*p* *	FLQ Score	*p* *	BRS Score	*p* *
Women (*N* = 405)	39 (35–43)	0.258	70 (63–76)	0.067	3.3 (2.8–3.7)	<0.001
Men (*N* = 115)	39 (34–44)		69 (63–75)		3.5 (3.2–4)	
Years of study: 1st to 3rd (*N* = 304)	39 (34–43)	0.152	69 (63–76)	0.476	3.3 (2.8–3.7)	0.928
Years of study: 4th to 6th (*N* = 216)	40 (36–44)		70 (64–75)		3.3 (2.9–3.8)	
Dental Medicine (*N* = 169)	39 (35–42) ^a^	0.002 ^§^	70 (62–76)	0.903 ^§^	3.3 (2.8–3.7)	0.382 ^§^
Medicine (*N* = 268)	38 (34–43) ^a^		69 (63–75)		3.5 (2.8–3.8)	
Pharmacy (*N* = 83)	42 (37–45)		68 (64–74)		3.3 (2.8–3.8)	
Good self-reported stress-coping ability (*N* = 380) ^†^	38 (34–42)	<0.001	71 (66–77)	<0.001	3.5 (3.2–3.8)	<0.001
Poor self-reported stress-coping ability (*N* = 140)	41 (38–46)		64 (58–69)		2.7 (2.2–3.2)	
Self-reported MHD history present (*N* = 159) ^‡^	42 (37–46)	<0.001	66 (59–72)	<0.001	3.0 (2.3–3.5)	<0.001
Self-reported MHD history absent (*N* = 361)	39 (34–42)		71 (66–76)		3.5 (3–3.8)	
Sought help (*N* = 44)	44 (38–49)	0.014	63 (56–70)	0.195	3.0 (2.2–3.4)	0.159
Did not seek help (*N* = 115)	40 (36–45)		67 (59–72)		3.2 (2.5–3.5)	
Confirmed MHD (*N* = 32)	43 (38–47)	0.339	61 (54–69)	0.074	3.0 (2.3–3.3)	0.439
Subjectively perceived MHD without official confirmation (*N* = 127)	41 (37–46)		67 (60–72)		3.2 (2.5–3.5)	

Data are presented as median (interquartile range); MHD—mental health disorder; BRS—Brief Resilience Scale; FLQ—FANTASTIC Lifestyle Questionnaire; OBI—Oldenburg Burnout Inventory; * Mann–Whitney U test; *p* values are unadjusted descriptive comparisons; ^†^ subjective self-assessment of stress-coping ability; ^‡^ self-reported MHD history present, defined as confirmed MHD diagnosis or subjectively perceived MHD without official confirmation; ^§^ Kruskal–Wallis test with post hoc Conover analysis; ^a^ Comparison with Pharmacy group (*p* < 0.05).

**Table 3:** Age- and gender-adjusted logistic regression model for self-reported MHD history being present.

Variable	Adjusted OR	95% CI	*p*
Female gender (vs. male)	1.513	0.894–2.562	0.123
Age (per year)	0.978	0.886–1.079	0.654
BRS total score (per 1-point increase)	0.539	0.389–0.747	<0.001
FLQ total score (per 1-point increase)	0.951	0.929–0.974	<0.001

BRS, Brief Resilience Scale; CI, confidence interval; FLQ, FANTASTIC Lifestyle Questionnaire; MHD, mental health disorder; OR, odds ratio. The model included age and gender as a priori covariates; BRS and FLQ scores were retained by forward selection. OBI score was evaluated but not retained.

**Table 4:** Sensitivity analyses separating confirmed and subjectively perceived MHD history.

Sensitivity Outcome	Variable	Adjusted OR	95% CI	*p*
Confirmed diagnosis vs. absent (*N* = 393; cases = 32)	BRS total score	0.554	0.302–1.017	0.057
Confirmed diagnosis vs. absent (*N* = 393; cases = 32)	FLQ total score	0.924	0.882–0.967	<0.001
Perceived unconfirmed vs. absent (*N* = 488; cases = 127)	BRS total score	0.538	0.380–0.762	<0.001
Perceived unconfirmed vs. absent (*N* = 488; cases = 127)	FLQ total score	0.955	0.931–0.980	<0.001

BRS, Brief Resilience Scale; CI, confidence interval; FLQ, FANTASTIC Lifestyle Questionnaire; MHD, mental health disorder; OR, odds ratio. Each sensitivity model was adjusted for age and gender. “Absent” denotes students without self-reported MHD history. The confirmed-diagnosis model excluded students with subjectively perceived but unconfirmed MHD history; the perceived-unconfirmed model excluded students with confirmed MHD diagnoses.

## Data Availability

The data presented in this study are available on request from the corresponding author due to privacy and ethical restrictions related to the sensitive nature of self-reported mental health data.

## Supplementary Materials

The following supporting information can be downloaded at: https://www.mdpi.com/article/10.3390/ejihpe16070089/s1, Table S1: Adjusted differences in questionnaire scores across demographic groups using Quade non-parametric ANCOVA (*N* = 520).

## Abbreviations

The following abbreviations are used in this manuscript:

Abbreviation	Full form
ANCOVA	Analysis of covariance
BRS	Brief Resilience Scale
CI	Confidence interval
COVID-19	Coronavirus disease 2019
FLQ	FANTASTIC Lifestyle Questionnaire
JD-R	Job Demands–Resources
MHD	Mental health disorder
OBI	Oldenburg Burnout Inventory
OBI-D	Oldenburg Burnout Inventory—Disengagement subscale
OBI-E	Oldenburg Burnout Inventory—Exhaustion subscale
OR	Odds ratio
WHO	World Health Organization
