# Supplementary material for: Self-Reported Mental Health History and Self-Reported Coping Behaviours in Biomedicine Students—Exploring Associations with Healthy Lifestyle and Resilience
Source: Eur J Investig Health Psychol Educ. 2026 Jun 29;16(7):89. doi: 10.3390/ejihpe16070089 (PMC13409207; doi:10.3390/ejihpe16070089)
Supplement: Supplementary file 1 [file ejihpe-16-00089-s001.zip › ejihpe-4328129-supplementary.pdf]

## Supplement

**Table S1.** Adjusted differences in questionnaire scores across demographic groups using Quade non-parametric ANCOVA (N=520).

| Grouping variable | Outcome | Covariates included in Quade ANCOVA | Test statistic | p value |
|---|---|---|---|---|
| Gender | OBI score | Age, BRS score, FLQ score | F(1,515)=0.579 | 0.447 |
| Gender | FLQ score | Age, BRS score, OBI score | F(1,515)=6.022 | 0.014 |
| Gender | BRS score | Age, FLQ score, OBI score | F(1,515)=23.248 | <0.001 |
| Study-year category | OBI score | Age, BRS score, FLQ score | F(1,515)=3.430 | 0.065 |
| Study-year category | FLQ score | Age, BRS score, OBI score | F(1,515)=8.215 | 0.004 |
| Study-year category | BRS score | Age, FLQ score, OBI score | F(1,515)=0.001 | 0.982 |
| Study programme | OBI score | Age, BRS score, FLQ score | F(2,514)=8.954 | <0.001 |
| Study programme | FLQ score | Age, BRS score, OBI score | F(2,514)=1.533 | 0.217 |
| Study programme | BRS score | Age, FLQ score, OBI score | F(2,514)=2.042 | 0.131 |

**Abbreviations:** BRS, Brief Resilience Scale; FLQ, FANTASTIC Lifestyle Questionnaire; OBI, Oldenburg Burnout Inventory.
